# Mandibular reconstruction using an axially vascularized tissue-engineered construct

**DOI:** 10.1186/1750-1164-5-2

**Published:** 2011-03-20

**Authors:** Ahmad M Eweida, Ayman S Nabawi, Mona K Marei, Mohamed R Khalil, Habashi A Elhammady

**Affiliations:** 1 Department of Head and Neck and Endocrine Surgery, Faculty of Medicine, University of Alexandria, Egypt; 2 Tissue Engineering Laboratories, Faculty of Dentistry, University of Alexandria, Egypt

## Abstract

**Background:**

Current reconstructive techniques for continuity defects of the mandible include the use of free flaps, bone grafts, and alloplastic materials. New methods of regenerative medicine designed to restore tissues depend mainly on the so-called *extrinsic neovascularization*, where the neovascular bed originates from the periphery of the construct. This method is not applicable for large defects in irradiated fields.

**Methods:**

We are introducing a new animal model for mandibular reconstruction using *intrinsic axial vascularization *by the Arterio-Venous (AV) loop. In order to test this model, we made cadaveric, mechanical loading, and surgical pilot studies on adult male goats. The cadaveric study aimed at defining the best vascular axis to be used in creating the AV loop in the mandibular region. Mechanical loading studies (3 points bending test) were done to ensure that the mechanical properties of the mandible were significantly affected by the designed defect, and to put a base line for further mechanical testing after bone regeneration. A pilot surgical study was done to ensure smooth operative and post operative procedures.

**Results:**

The best vascular axis to reconstruct defects in the posterior half of the mandible is the facial artery (average length 32.5 ± 1.9 mm, caliber 2.5 mm), and facial vein (average length 33.3 ± 1.8 mm, caliber 2.6 mm). Defects in the anterior half require an additional venous graft. The defect was shown to be significantly affecting the mechanical properties of the mandible (P value 0.0204). The animal was able to feed on soft diet from the 3^rd ^postoperative day and returned to normal diet within a week. The mandible did not break during the period of follow up (2 months).

**Conclusions:**

Our model introduces the concept of axial vascularization of mandibular constructs. This model can be used to assess bone regeneration for large bony defects in irradiated fields. This is the first study to introduce the concept of axial vascularization using the AV loop for angiogenesis in the mandibular region. Moreover, this is the first study aiming at axial vascularization of synthetic tissue engineering constructs at the site of the defect without any need for tissue transfer (in contrast to what was done previously in prefabricated flaps).

## Background

Bone tissue has regenerative capabilities that enable the self-repair of fractures and tissue loss; however, in extreme situations in which the extent of bone loss or damage due to trauma, surgery, or a metabolic disease is too large, spontaneous complete regeneration cannot occur.

Traditionally, the augmentation of bony defects is carried out using allografts, xenografts, autogenous bone, and synthetic biomaterials. The transplantation of autogenous bone is regarded as the gold standard. Globally, there are more than 2 million autogenous bone transplantations in humans each year [1,2]. Because of the osteoinductive and osteoconductive characters [3] of autogenous bone, there are a number of good results obtained upon transplantation. However, there are disadvantages, namely:

1. In most cases, two surgical procedures are necessary: one for bone harvesting (e.g., from the iliac crest) and the other for implantation. This can cause some patients to suffer from complications associated with the donor site.

2. At the site of bone transplantation, the risks of wound infection, necrosis, and resorption, representing up to 30% of transplanted material, have been experienced [1,2].

One major reason for mandibulectomy and maxillectomy is oral cancer; an estimated 34,000 of such cases were anticipated in the United States in 2008 [4]. In addition, approximately 1,600,000 bone grafts are performed each year to regenerate bone lost due to trauma or disease, of which 6% (96,000) are craniomaxillofacial in nature [5]. Current reconstructive techniques for continuity defects of the mandible encompass the use of revascularized free flaps, free nonvascularized bone grafts, and alloplastic materials. Although revascularized composite flaps may be regarded as the "gold standard," they are associated with donor site morbidity [6,7].

Tissue engineering methods designed to restore diseased and damaged tissues depend on the presence of a matrix structure (scaffold) that is amenable to cell growth and proliferation. In order to support the development of complex biologic structure, such biomaterials must effectively interact with the surrounding tissue and incite the host to populate the graft with new tissue. To accomplish this task, these matrices must either create-or induce the host to establish an early and aggressive angiogenic response leading to the development of a blood supply for the restoration of structure and function [8].

The majority of currently applied tissue-engineering approaches rely on the so-called ***extrinsic mode of neovascularization***. In this setting, the neovascular bed originates from the periphery of the construct, which should be implanted into a site of high vascularization potential [9]. This represents a major obstacle for bone regeneration in post irradiated regions. Furthermore, diffusion limits oxygen and nutrition supply to cells to a maximum range of 200 μm into a given matrix. That is why suboptimal initial vascularization often limits survival of cells in the center of large constructs [10]. These issues of vascularization implemented the need for novel angiogenic approaches and new in vivo models evolved with the aim to generate constructs with ***a dedicated neovascular network ***not under the immediate influence of the local environment [11]. In 1979 Erol and Spira reported about their work on vascular induction by means of inserting microvascular constructs onto free skin grafts. Several vessel configurations were investigated including a flow through vascular pedicle, a distally ligated arteriovenous pedicle as well as an arteriovenous (AV) fistula. The latter was found to possess the highest capacity of inducing and sustaining vascularization into the free skin transplant. As a result, a new tissue element was generated with a dedicated vascular network based on an arteriovenous axis. The axial vascularization of the new flap was similar to the pattern seen in tissue transplants suitable for microvascular transfer (free flaps). During the late 1980s the principle was refined and found a way into plastic surgical reconstruction under the collective designation of the so called 'prefabricated free flaps' [12]. Recently, the superiority of the AV-loop as a vascular carrier for an axial type of vascularization has been clearly demonstrated [9,13,14].

We are introducing here a new animal model for mandibular reconstruction using *intrinsic axial vascularization *by the AV loop. In order to test this model, we have performed cadaveric, mechanical loading, and surgical pilot studies on adult male goats.

## Methods

Our model is based on Cadaveric studies, mechanical loading studies, and pilot surgical studies. Qualitative and quantitative data on angiogenesis and osteogenesis, together with the long term follow up of this model are being further studied by our team.

### • Cadaveric studies

We studied the mandibular and parotid regions in the goat aiming at good orientation of the anatomy, specially the vascular anatomy. Our goal was identification of the best vascular axis to be used in creating the AV loop. The study included 6 fresh cadaveric adult male goat heads; 3 right and 3 left.

### • Mechanical loading studies and design of the defect

**Figure 1:**
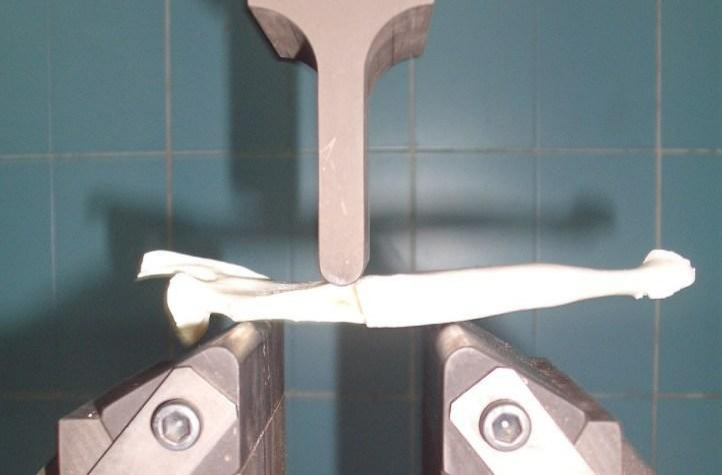
**Three points bending test applied on the goat mandible**.

We studied the mechanical loading of the normal goat mandible aiming for detecting reference levels for the 'break point' at the region of the angle of the mandible. The Equipment used is the 3 Points Bending apparatus; Autograph AG-IS 100 KN, SHIMADZU (Mubarak City for Scientific Research, Borg El-Arab). Four adult male goat mandibles where sharply dissected from cadavers and the large soft tissue parts were removed by sharp dissection. Each mandible was submerged in 30% Hydrogen peroxide for 5 minutes. The residual soft tissue was mechanically removed under running tap water. The apparatus was adjusted so that the 2 resting points where 8 cm apart. The mandible was placed horizontally on the 2 resting points so that the lateral side of the mandible is facing upwards (towards the pressing blade). The mandible was slightly tilted so that the pressing blade will apply the load on a line overlying the anterior boundary of the designed defect (figure 1). The rate of application was adjusted to 1 mm/minute. The results were plotted in the form of graphs where the force (N) is plotted against the stroke (mm). The data was calculated as mean value ± standard deviation (SD) and analyzed using the Graphpad online software.

**Figure 2:**
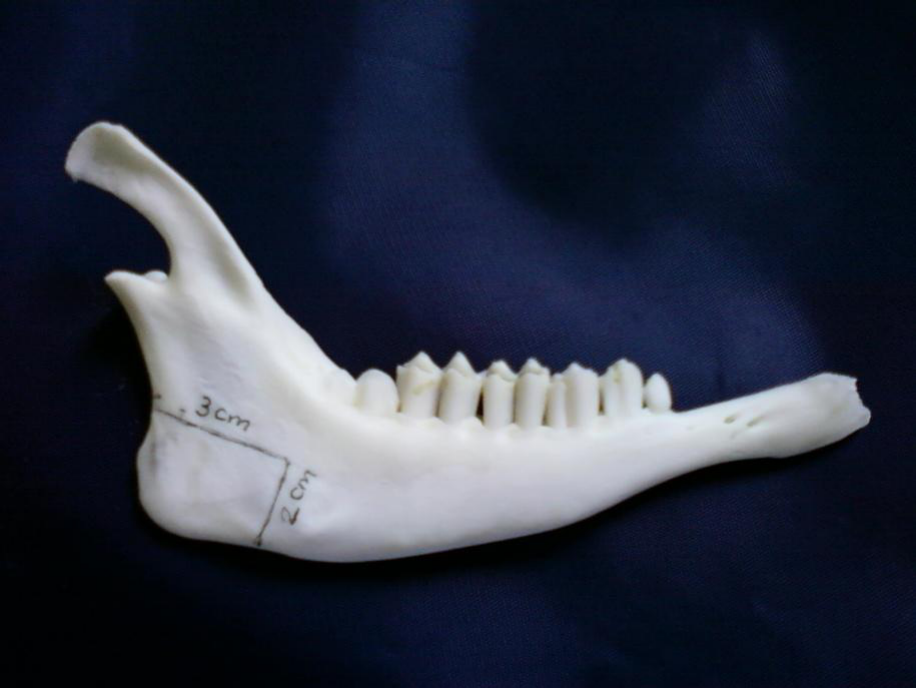
**Design of the defect**.

**Figure 3:**
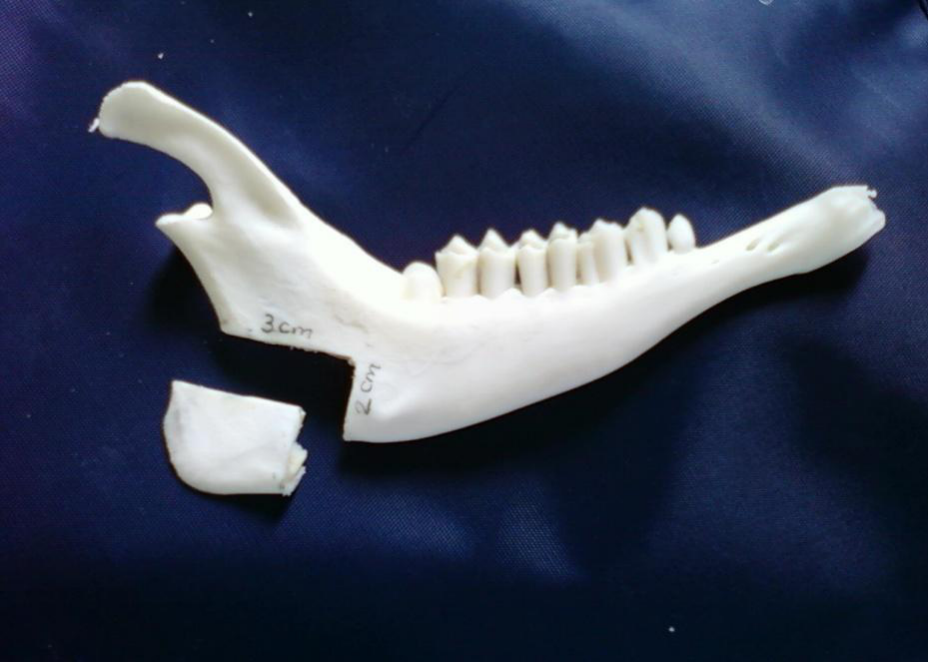
**The defect created**.

In order to detect the 'break point' after creating the defect at the angle of the mandible, another four adult male goat mandibles were dissected and prepared as previously mentioned. A 3 × 2 cm full-thickness rectangular defect was created at the angle of the mandible. The anterior border of the defect (2 cm) coincides with a line drawn between the notch at the posterior lower margin of the mandible (constant bony landmark) and the posterior edge of the last molar. (figures 2,3). The defect was created using an oscillating saw (5400-031 Stryker TPS, USA). The 3 points bending test was done for the resected mandibles as previously mentioned.

### • Animal Surgery

In order to assess the feasibility of our model, and to insure smooth intra and post operative procedures, we performed a pilot animal surgery on a 3 year-old male goat. All the procedures were done according to the NIH guidelines for animal surgery [15]. The animal fasted overnight. General IM anesthesia was induced using Xylazine HCl 0.22 mg/kg. 15 minutes later ketamine HCl IM was administrated at a dose of 11 mg/kg. The dose of Ketamine was repeated twice throughout the operation (every 25 minutes).

#### Procedure

**Figure 4:**
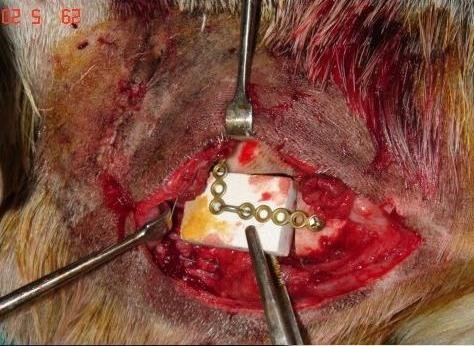
**The scaffold placed in the mandibular defect**.

**Figure 5:**
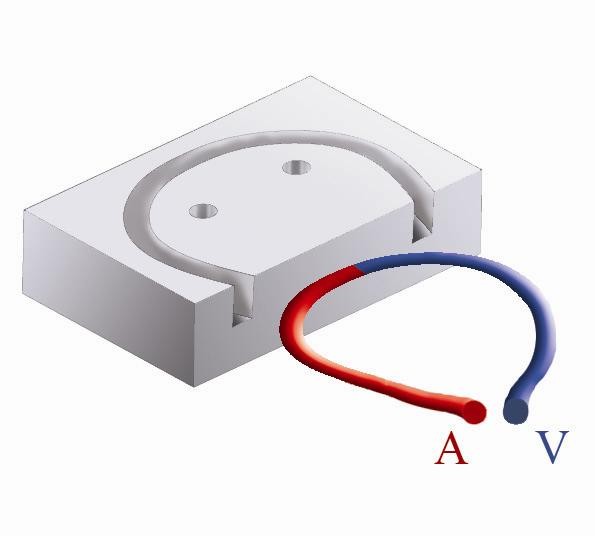
**Diagram showing how the AV loop will be laid inside the scaffold**.

**Figure 6:**
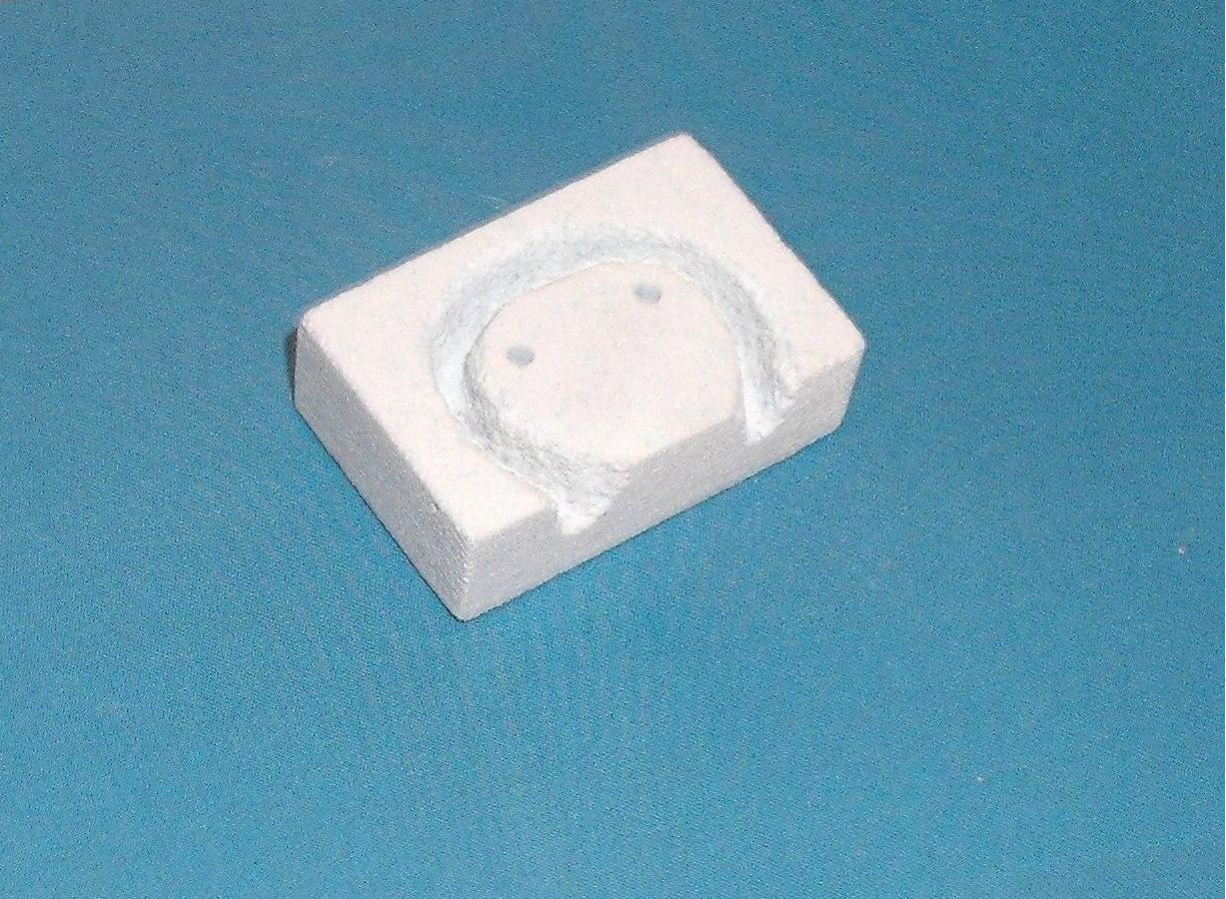
**The grooved scaffold**.

In a left lateral decubitus with head extended, the right mandibular region was shaved and disinfected using povidine iodine (Betadine). Dissection was done in layers through a submandibular skin incision 10 cm long. The periosteum covering the angle of the mandible was elevated together with the masseter muscle, parotid gland and fascia using sharp dissection and a periosteal elevator. Using the oscillating saw (5400-031 Stryker TPS, USA) a 3 × 2 cm full-thickness defect was created at the angle of the mandible as previously designed. Continuous irrigation with normal saline 0.9% is done throughout the sawing procedure. The bone segment was sharply dissected from the underlying medial pterygoid muscle. Small pieces of Gelatin sponge (Gelfoam, Pfizer) and Oxidized regenerated cellulose (Surgicel, Ethicon Inc.) were used for hemostasis of the field before application of the scaffold. The preformed scaffold (60% HA, 40% βTCP, 62% porosity, BioGraft Dental Bone Granules and Blocks, ISO 9001:2000, CE Certifications-CE 1023) was mounted to a titanium miniplate (4 × 5 holes) using a stainless steel wire as shown (figure 4). Two 2 mm screws were used to fix the miniplate to the mandible. The facial artery and vein could be easily anastomosed using 9/0 polypropylene sutures (Prolene, Ethicon) and laid into a deep groove in the scaffold (reaching 5 mm depth in the 10 mm-thick scaffold), covered with granules of the same material and kept in place by the titanium plate (figures 5, 6). The masseter muscle and parotid fascia were sutured back to cover the defect using Vicryl 1/0 continuous sutures. The skin was closed using interrupted simple 1/0 Silk sutures. The wound was disinfected with povidine iodine (Betadine) and left uncovered. 30 mg ketorolac tromethamine (Ketolac) IM was given immediately postoperative and once daily for 2 days for analgesia. Oxytetracycline trihydrate 250 mg IM was administrated once daily for 2 days postoperatively as prophylactic antibiotic. During the first postoperative day the animal received NPO and 500 cc glucose 5%. During the second day the animal received 500 cc glucose 5% and free fluid intake was allowed. From the 3^rd ^day till the end of the first week, free fluid intake and soft diet with supplements were allowed. The animal returned to normal diet after one week.

## Results

### • Cadaveric study

**Figure 7:**
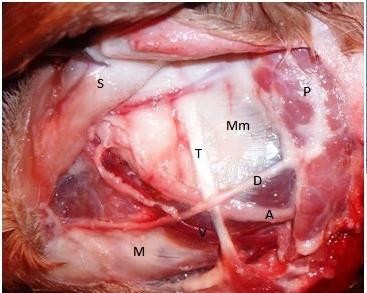
**Lt. Parotid region**. S: Reflected skin, Mm: Masseter muscle, T: tendon of Longissimus capitis muscle, P: Parotid gland, D: Duct of parotid gland, M: Body of the Mandible, V: Facial vein, A: Facial artery.

**Figure 8:**
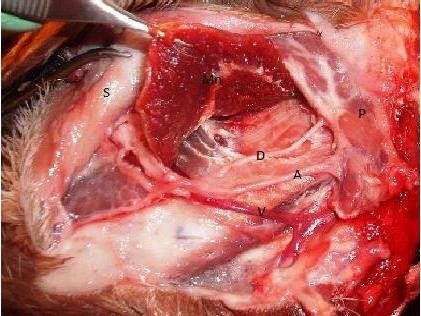
**Lt. Parotid region**. S: Reflected skin, Mm: Reflected Masseter muscle, P: Parotid gland, D: Duct of parotid gland, V: Facial vein, A: Facial artery

The vessels available in the mandibular region are the facial, transverse facial and lingual vessels. The best vascular axis to be used is the facial artery and facial vein. The lingual and transverse facial vessels are much smaller, far from the mandible, and their dissection requires disturbance of the parotid gland. Moreover, the diameter of the facial vessels was more or less constant within the parotid region due to fine branching that did not significantly affect the vessel caliber. The facial artery arises from the External carotid artery (via linguofacial trunk) and the facial vein unites with the lingual vein to form the linguo-facial vein to drain into the Jugular vein. The facial vessels run epifascially parallel to each other in an angle of 45° to the long axis of the mandible. The artery is cranial to the vein (5 mm apart). Both vessels are deep to the tendon of longissimus capitis muscle and the duct of the parotid gland. The average length of the facial artery from the anterior margin of the parotid gland till the alveolar margin is 32.5 ± 1.9 mm. The length of the facial vein from the alveolar margin till uniting with the lingual vein is 33.3 ± 1.8 mm. The average diameter of the facial artery is 2.5 mm and the facial vein is 2.6 mm. The anastomosis can be easily done in the region of the anterior mandible using the surgical loupe or operative microscope (figures 7,8).

### • Mechanical loading studies

**Figure 9:**
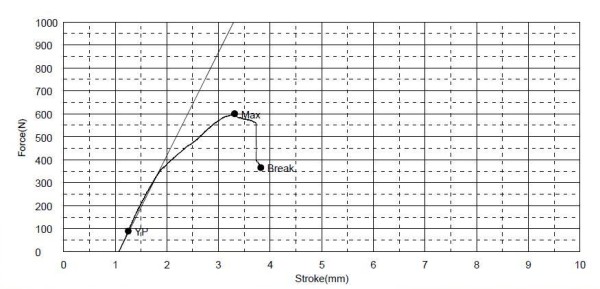
**A graph showing the results of 3 points bending test on a normal mandible**.

**Figure 10:**
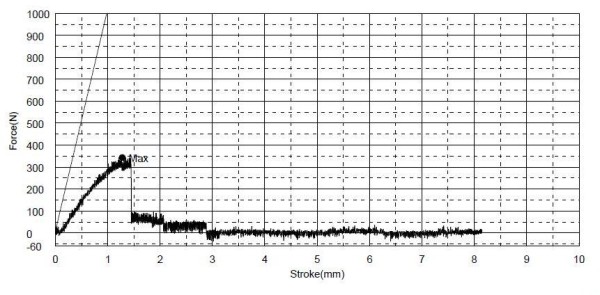
**A graph showing the results of 3 points bending test on a resected mandible**.

The study showed the feasibility of using the 3 points bending test for mechanical loading analysis at the region of the angle of the mandible in this model. The *average *force used to break the normal mandible was 640.2 ± 137.89N (Figure 9). The inner (medial) table of the mandible broke before the outer (lateral) table and the line of break passed close to the alveolar foramen while the line of break of the outer table is the line of application of the force. The average force used to break the mandible with defect was 210.63 ± 108.92 N (Figure 10). The sequence and line of break was similar to that of the normal mandible. The two-tailed P value equals 0.0204 and is considered to be statistically significant.

### • Animal surgery

The study showed the feasibility of the surgical procedures to be done on the goat model under general anesthesia with minimal animal morbidity. The animal was able to feed on soft diet from the 3^rd ^postoperative day and returned to normal diet in a week postoperatively. The mandible did not break during the period of follow up (2 months).

Based on the cadaveric, mechanical, and surgical studies, this model can be standardized as a safe efficient model for studying axially vascularized mandibular regeneration in large animals. The next step will be charging the scaffold with suitable growth factors e.g. BMP2 and establishment of the AV loop inside the scaffold then investigating the resulting angiogenesis and osteogenesis.

## Discussion

Radiotherapy is commonly used as primary treatment and as an adjuvant to the surgical excision of malignant tumours especially in the head and neck regions. While radiotherapy is an effective post operative measure in destroying potential residual cancer cells, its side effects are well-documented and include damage to normal epithelial, dermal, and endothelial cells [16]. The resulting hypocellularity and hypoxic environment leads to scarring and fibrosis that make secondary reconstruction of the surgical site difficult. This further adds to the challenges against reconstruction using the modalities of regenerative medicine or tissue engineering. Regeneration at the central region of large constructs usually fails due to absence of adequate vascularization [10].

One tissue-engineering approach to the regeneration of bone involves the delivery of cell signaling factors such as growth factors or genes from biomaterial scaffolds. Growth factors such as VEGF, TGF-β1, and BMPs have been delivered to defects and have significantly improved bone repair in irradiated sites [17-21]. Other research groups tried sustainably expressing osteoinductive factors through cells that are transduced *in vivo *[22] in regenerating defects compromised by radiotherapy [23]. However, these studies did not demonstrate clinically relevant complete regeneration of critical-sized defects in large animals. Furthermore, large doses are often necessary in protein-based therapies, which are extremely expensive and thus may be impractical for universal clinical application.

Other research groups started to address the issues of vascularization in order to be able to deliver clinically-relevant sized tissues with axial vascularization to the site of reconstruction [24-26].

The sheep groin AV-loop model was the very first large animal model for de novo creation of axially vascularized tissue using microsurgical techniques [27]. That model was further standardized and axial vascularization was successfully induced in a large volume (16 cc) of a clinically approved biphasic calcium phosphate ceramic [28]. Yet, all these trials aimed at axially-vascularizing constructs that would be later on transferred to the site of reconstruction.

Successful trials for mandibular reconstruction in clinically-relevant sized defects using the issues of axial vascularization have not been reported. The concept of axial vascularization of bones was previously introduced in research studies [29] and even in clinical practice in order to revascularize bones as in cases of avascular necrosis of scaphoid bone or Kienboeck's disease. These methods used either ligated vascular pedicles or AV loops with or without bone grafting [30,31]. However, the concept of induction of axial vascularization of synthetic tissue engineering constructs at the very same site of reconstruction without the need for tissue transfer (with subsequent operative and microsurgical hazards accompanying tissue transfer) has not been introduced.

Our concept regarding axial vascularization of mandibular constructs is based on solid data in literature confirming the efficiency of using the AV loop model for axial vascularization of transplantable tissues [12,13,32]. In order to further evaluate this in the mandible, we introduced a new design of a mandibular defect in goats. The defect should have a critical size [33], and should significantly affect the mechanical properties of the mandible. However, the defect design should be as simple as possible in order not to add to the complexity of the procedure which involves micro-anastomosis inside the construct. The model aimed at minimizing the animal morbidity where there is no loss of mandibular continuity and intact oral mucosa.

Our studies showed that direct anastomosis of facial vessels allows the AV loop to be set in for reconstruction of defects in the posterior half of the mandible. Defects in the anterior half, however, may require a venous graft between the facial artery and vein. The caliber of the facial vessels can allow anastomosis to be done easily using the surgical loupe or operative microscope.

Regarding the status of the mandibular region after cancer surgery and irradiation, our AV loop model can be quite suitable for further clinical applications. In most of the cases of head and neck cancers requiring mandibulectomy the facial vessels could be preserved. Being medium sized vessels, the facial vessels can be used even after exposure to irradiation as recipient vessels for free flaps [34] and thus can be technically suitable to construct the AV loop.

The study showed that the 3 points bending mechanical test is a simple and feasible method for testing the region of the angle of the mandible in our defect model. Although the test does not mimic the normal physiological stresses on the goat mandible, which is very difficult to analyze and simulate, it is efficient in detecting the difference in the strength between the normal and the resected mandible. This difference was shown to be significant. Applying this test on the regenerated mandible after explantation will provide information about the mechanical properties of the regenerated bone in the mandible in comparison to the normal and the resected mandibles.

Our pilot study showed the feasibility of the surgical procedures to be done on the goat model under general anesthesia with minimal animal morbidity.

## Conclusions

We introduced a model for axial vascularization of a tissue -engineered mandibular construct using the AV loop. To the best of our knowledge, this is the first study to introduce the concept of axial vascularization using the AV loop for angiogenesis in the mandibular region. Moreover, this is the first study aiming at axial vascularization of a synthetic tissue engineering construct at the same site of the defect without any need for tissue transfer (in contrast to what was done previously in prefabricated flaps). Qualitative and quantitative data on angiogenesis and osteogenesis, together with the long term follow-up of this model is now being further studied by our team.

## Competing interests

The authors declare that they have no competing interests.

## Authors' contributions

AME designed the study, performed the cadaveric study, carried out the surgical operations, and drafted the manuscript. ASN participated in the surgical design and operations. MKM helped with the surgical operations. MRK supervised the study design. HAE supervised the study and the surgical operations. All the authors approved the final manuscript.
