# Correction: Mandibular reconstruction using an axially vascularized tissue-engineered construct

**DOI:** 10.1186/1750-1164-6-4

**Published:** 2012-06-15

**Authors:** Ahmad M Eweida, Ayman S Nabawi, Mona K Marei, Mohamed R Khalil, Habashi A Elhammady

**Affiliations:** 1 Department of Head and Neck and Endocrine Surgery, Faculty of Medicine, University of Alexandria, Alexandria, Egypt; 2 Tissue Engineering Laboratories, Faculty of Dentistry, University of Alexandria, Alexandria, Egypt

## Correction

After publication of this work [1], the authors noted that there were some corrections that should be added to the work. These corrections are done in order to modify some parts that were unfortunately missed during the original publishing procedures. The authors would like to apologize for the inconvenience.

Page 2 in the Methods section under “Mechanical loading studies and design of the defect” line 13: “4 cm” should be written instead of “8 cm.”

In the legends of Figures 7 and 8, the right tag should be as follows: A: Parotid duct, D: Facial nerve, V: facial vessels.

## Competing interests

The authors declare that they have no competing interests.
